# Effect of 2 Bleaching Agents with a Content of High Concentrated Hydrogen Peroxide on Stained 2 CAD/CAM Blocks and a Nanohybrid Composite Resin: An AFM Evaluation

**DOI:** 10.1155/2017/6347145

**Published:** 2017-07-18

**Authors:** İzgen Karakaya, Esra Cengiz

**Affiliations:** ^1^Department of Restorative Dentistry, Faculty of Dentistry, Near East University, Northern Cyprus, Mersin 10, Turkey; ^2^Department of Restorative Dentistry, Faculty of Dentistry, Mersin University, Mersin, Turkey

## Abstract

The aim of this study was to evaluate color stability of 3 restorative materials, discoloration ability of different solutions, efficacy of 2 office bleaching agents, and surface roughness and topography. Sixty specimens for Clearfil Majesty Esthetic (CME), Lava Ultimate (LU), and Vita Enamic (VE) were prepared. They were immersed into 3 staining solutions for 2 weeks and then they were bleached. According to the measured *L*^⁎^, *a*^⁎^, and *b*^⁎^ parameters described by CIELAB system, color changes (Δ*E*_00_), translucency parameters (TP), whiteness index values (*W*^⁎^), and changes in closeness to pure white (Δ*W*^⁎^) were calculated. Then 3 specimens from each group were scanned with an atomic force microscope for surface analysis. After staining, CME groups and control groups of LU and VE showed clinically acceptable color changes (Δ*E*_00_ < 1,8). After bleaching, while a reverse effect on color was observed, VE showed the furthest color values to pure white. There was no statistically significant difference between whiteness index values of LU and CME. LU was the most translucent material during the study and TP values of materials showed minimal differences. Most of the VE groups and a control group of LU showed surface roughness (*R*_*a*_) values higher than critical value for biofilm accumulation (0,2 *μ*m).

## 1. Introduction

Esthetic has been one of the most challenging factors for restorative dentistry. Regarding the general changes in the definition of the esthetical appearance in dentistry as healthy, natural, beautiful, and confident smiles, bleaching and the smile designing procedures have been too popular over a few decades [1]. Therefore, there has been an accelerated development of dental materials and techniques such as bleaching agents, tooth colored restorative materials, and especially Computer Aided Design and Computer Aided Manufacturing (CAD/CAM) technology and CAD/CAM blocks [2–4].

During the passing time, esthetic qualities (color, chromaticity, and translucency) and mechanical properties (strength, wear resistance, and water sorption) of composite resin materials have been improved by the changes of the chemical structure of organic matrix and particle sizes and quantities of fillers [5, 6]. Thus, it became possible to use composite resins for both anterior and posterior, direct, and indirect restorations [5, 6]. Recently, nanofilled or nanohybrid composite resins are the most preferred resin materials [7]. Nanohybrid composite resins contain 0,01–0,04 nm sized and clustered agglomerate fillers that may lead to increased filler content, reduced polymerization shrinkage, increased wear resistance, better surface smoothness, polychromacity, multitranslucency, and gloss retention [6, 8]. In spite of all the recent advances and developments in composite resin materials, the most important problem is still polymerization shrinkage [9, 10].

To overcome the drawbacks like polymerization shrinkage, monomer release, porosities, inhomogeneity, and color stability problems at direct composite restorations, the combination of composite resins, ceramics, and CAD/CAM technology as ceramic/nanoceramic hybrid CAD/CAM blocks has been introduced [4, 6, 11, 12]. The disadvantages of conventional and milled ceramic restorations by CAD/CAM like necessity of more preparation and more steps related to both clinical and laboratory procedure, difficulties in the repair of the restoration, and lack of proper esthetic properties and monochromatic appearance have prompted the use of ceramic/nanoceramic hybrid CAD/CAM blocks [6, 11, 12].

Office bleaching is a method applied by professionals to solve the discoloration of the teeth related to intrinsic and extrinsic factors [13–15]. This method generally uses high concentrations of hydrogen peroxide (HP) which is leading to the oxidation of the pigments present in enamel and dentin as a cause of its low molecular weight and free transit through the interprismatic spaces of enamel and throughout dentin [13–15]. Although bleaching is accepted as an effective method and safe for hard tissues of teeth, it may not be safe for dental materials that have high erosive or degradation characteristics [16]. The chemical reactions between bleaching agent and dental materials may cause alterations on the surface topography [17–19]. Due to the structural differences, ceramic/nanoceramic hybrid CAD/CAM blocks are expected to show different resistance to the bleaching agents compared to the composite resins.

The effects of the bleaching agents on composite resins have been investigated by several studies [16–22]; however, to the best of our knowledge, the available data regarding the effect of office bleaching on the color of CAD/CAM blocks is limited. Therefore, the aim of this study was to evaluate color stability of a composite resin and 2 CAD/CAM blocks, discoloration ability of different solutions, and effect of 2 office bleaching agents with different concentrations of HP on color, translucency, and the surface topography of stained restorative materials. The null hypotheses tested in this study were as follows: (1) both the composite resin and the CAD/CAM blocks have good color stability after storage in staining solutions, (2) the bleaching agents can provide a reverse effect on color of stained composite resin and CAD/CAM blocks, (3) staining and bleaching will not affect the translucency parameter (TP), and (4) staining and bleaching will not affect the surface topography of the materials tested.

## 2. Materials and Methods

The materials tested in this study were a nanohybrid composite resin (Clearfil Majesty Esthetic [CME]) and 2 ceramic/nanoceramic hybrid CAD/CAM blocks (Lava Ultimate [LU], Vita Enamic [VE]). The compositions and manufacturers of the materials were presented in Table 1.

### 2.1. Specimen Preparation

For A2 shaded composite resin, 60 disc shaped specimens were prepared by using a Teflon mold with 1 mm thickness and 10 mm diameter. The mold was placed between 2 glass slides, covered with a transparent polyester strip which were gently pressed together to remove excess composite resin. Both of the top and bottom surfaces of the specimens were polymerized by a conventional halogen curing light (Hilux Ultra Plus, Benlioğlu Dental Inc., Ankara, Turkey) with a light intensity of 600 mW/cm^2^ for 40 seconds. Then, composite discs were kept in distilled water at 37°C for 24 hours to ensure the completion of polymerization. At the end of 24 hours, the top surfaces of the specimens were polished by using coarse, medium, fine, and superfine Sof-Lex™ Discs (3M ESPE, St. Paul, MN, USA), respectively. For each A2 shaded CAD/CAM block, 60 rectangular shaped specimens with 1,3 mm thickness, 6 (±0,3) mm width and 7 (±0,3) mm length were prepared by using a low-speed diamond saw (Micracut 201, Metkon®, Bursa, Turkey) to supply the same surface area with composite resin materials. Specimens were polished on a wet polishing wheel with a sequence of 1200, 1500, and 2000 grit silicon carbide grinding papers. At the end of the polishing, thickness of the specimens was measured as 1 ± 0,1 mm and it was checked out by using a digital caliper (N48AA, Maplin Electronics, UK).

### 2.2. Immersion in Staining Solutions

The polished specimens were immersed in 20 ml distilled water at 37°C for 24 hours. At the end of 24 hours, all of the specimens were dried by using blotting paper and air flow. The baseline color measurements were performed by using a calibrated spectrophotometer (Vita Easyshade Compact, Vita Zahnfabrik, Bad Säckingen, Germany) at “tooth single” mode in a custom made viewing booth with D65 illumination (Master TL-D 90 De Luxe 18 W/965 1SL, Philips, Eindhoven, Holland). The probe of the spectrophotometer with a diameter of 5 mm was settled at the center of the specimens and *L*^*∗*^, *a*^*∗*^, and *b* parameters were recorded. “*L*^*∗*^ (the coordinates between white and black)” is the lightness component of the color; “*a*^*∗*^ (the coordinates between red and green)” and “*b*^*∗*^ (the coordinates between yellow and blue)” are the chromatic components of the color. *L*^*∗*^, *a*^*∗*^, and *b*^*∗*^ parameters were measured and recorded 3 times for each specimen on nonreflective white (*L*^*∗*^ = 96,3, *a*^*∗*^ = 0,1, and *b*^*∗*^ = 1,9) and black (*L*^*∗*^ = 8,9, *a*^*∗*^ = −0,7, and *b*^*∗*^ = 1,2) surfaces. Then the mean values of *L*_*w*_^*∗*^, *a*_*w*_^*∗*^, *b*_*w*_^*∗*^ and *L*_*B*_^*∗*^, *a*_*B*_^*∗*^, *b*_*B*_^*∗*^ were calculated where subscript “*W*” refers to the color coordinates measured on white surface and subscript “*B*” refers to the color coordinates measured on black surface. After each 9 measurements, calibration was repeated according to the manufacturer's instructions.

After baseline color measurements were completed, the specimens of each material were randomly divided into 3 subgroups according to the staining solutions. The specimens were immersed in 20 ml Turkish coffee (TC) (30 gr Turkish coffee boiled in 600 ml water, cooled, and filtered), 20 ml red wine (RW), or 20 ml distilled water (DW) (control) for 30 minutes per day. After 30 min, the specimens were taken out of the staining solutions, washed with distilled water, and dried, and for the remaining time of 24 hours all of the specimens were embedded in 20 ml distilled water. In a pilot evaluation [21], which is performed to establish the immersion time, optimal contact time in mouth for a hot beverage was reported as 60 seconds for each cup. Therefore, to simulate a total of 1 year with an average of 35 cups/glasses of beverage consumption per month, staining procedures were continued for consecutive 14 days.

### 2.3. Bleaching Procedure

In this study, Perfect Bleach Office+ (VOCO GmbH, Cuxhaven, Germany) with 35% HP and Opalescence Boost (Ultradent Products Inc., South Jordan, UT, USA) with 40% HP were used as the office bleaching agents. At the end of the 14th day of staining, all of the specimens were taken out of the distilled water and dried by using blotting paper and air flow. Following the color measurements, the bleaching agents were applied approximately 1 mm to the one surface of each specimen. While Perfect Bleach Office+ (PBO) was applied for 15 min, Opalescence Boost (OB) was applied for 20 min and they were activated by a microbrush at every 5 min according to the manufacturers' instructions. At the end of the time required for the bleaching procedure, the specimens were washed with a high pressure water flow and dried with blotting paper and air flow.

### 2.4. Color and Translucency Measurements

All of the color measurements were performed before the staining, at the end of 14th day of the staining and after the bleaching procedures. For color change after staining, Δ*E*_00_ (color difference) values were calculated with the following equation by using the recorded and calculated mean values of *L*_*w*_^*∗*^, *a*_*w*_^*∗*^, *b*_*w*_^*∗*^ and *L*_*B*_^*∗*^, *a*_*B*_^*∗*^, *b*_*B*_^*∗*^:

(1) ΔE00=ΔLkLSL2+ΔCkCSC2+ΔHkHSH2+RTΔCkCSCΔHkHSH1/2.

In this equation, Δ*E*_00_ is defining the color difference between baseline and after staining measurements. Δ*L*, Δ*C*, and Δ*H* are the differences in lightness, chroma, and hue between baseline and subsequent color measurements. *R*_*T*_ (rotation function) is a function that accounts for the interaction between chroma and hue differences in the blue region. *S*_*L*_, *S*_*C*_, and *S*_*H*_ are weighting functions for adjustment of the total color difference for variation in perceived magnitude with variation in the location of the color coordinate difference between 2 color measurements. *K*_*L*_, *K*_*C*_, and *K*_*H*_ as parametric factors are correction terms for experimental conditions. For the present study, the computation used for the Δ*E*_00_ formula was CIEDE2000 (1 : 1 : 1), where *K*_*L*_ = 1, *K*_*C*_ = 1, and *K*_*H*_ = 1 [23]. In this study, 50% : 50% perceptibility threshold for Δ*E*_00_ values was taken as 0,8 and Δ*E*_00_ > 0,8 was considered as a perceptible color difference [23]. 50% : 50% acceptability threshold was taken as 1,8 for Δ*E*_00_ values and the Δ*E*_00_^*∗*^ > 1,8 was considered as clinically unacceptable [23].

Translucency parameter (TP) was calculated with the following equation at baseline (TP0), after staining (TP1) and after bleaching procedures (TP2):

(2) TP=LB1∗−LW1∗2+aB1∗−aW1∗2+bB1∗−bW1∗21/2,

where TP is defining the translucency of the material. TP changes between 0 and 100 and if the values get closer to 100, it means increased translucency [21]. On the other hand, if TP gets closer to 0, it means increased opacity.

In order to quantify the whiteness before and after bleaching, the whiteness index (WIC index) which was recommended by CIE was used [15]. The whiteness index value (*W*^*∗*^) is based on the distance of a color value from a nominal white point, represented in CIELAB color space as *L*^*∗*^ = 100, *a*^*∗*^ = 0, and *b*^*∗*^ = 0, and defined according to the following equation:

(3) $$\begin{array}{c} W^{*}={\left[\;{\left(\;a^{*}\;\right)}^{2}+{\left(\;b^{*}\;\right)}^{2}+{\left(\;L^{*}-100\;\right)}^{2}\;\right]}^{1/2}. \end{array}$$

The nominal white point in the WIC index is equal to zero. Thus, the closer *W*^*∗*^ is to zero, the closer it is to pure white. In the present study, *W*^*∗*^ values were measured for the baseline (*W*0), at the end of the staining period (*W*1) and after bleaching (*W*2). Additionally, to whiteness index values, changes in closeness to pure white (Δ*W*^*∗*^) values were calculated by using the following equations:

 
Δ*W*_*a*_ = *W*2 − *W*0 (changes between baseline and after bleaching).
 
Δ*W*_*b*_ = *W*2 − *W*1 (changes between after staining and after bleaching).

A negative Δ*W*^*∗*^ indicates color coordinates that are closer to pure white and thus represents more favorable whiteness [15].

### 2.5. Scanning with Atomic Force Microscopy

Three specimens of each group were chosen randomly for surface roughness and surface topography analysis with an atomic force microscope (XE-100E, Park Systems, Induspia 5F, SangDaewon-Dong 517-13 Sungnam, Republic of Korea). The topography of the specimens was examined within an area of 30 × 30 *μ*m^2^ with a tip working in contact mode with an average scan rate of 0,3 Hz. The average *R*_*a*_ values of each specimen for surface roughness analysis were calculated by using measured average *R*_*a*_ values of 6 different linear places on scanned area. The acceptability threshold of surface roughness was chosen as 0,2 *μ*m [19]. *R*_*a*_ ≤ 0,2 *μ*m means minimal biofilm accumulation.

### 2.6. Statistical Analysis

Descriptive statistics were performed for each group and distribution of Δ*E*_00_, *W*, Δ*W*, TP, and *R*_*a*_ values was checked by the normality tests (Kolmogorov Smirnov for* W*0 and TP0, Shapiro-Wilk for the other values). All of the statistical analyses were performed by using software (SPSS Version 18, SPSS Inc., Chicago, IL, USA). Kruskal Wallis test was performed to check statistical differences between the TP0 and* W*0 values. For pairwise comparisons, Mann–Whitney* U* tests were applied.

Two-way ANOVA was performed for statistical analyses of Δ*E*_00_, *W*1, and TP1 values. The Tukey test was applied for pairwise comparisons with 95% confidence intervals. Three-way ANOVA was performed for *W*2, Δ*W*_*a*_, Δ*W*_*b*_, TP2, and *R*_*a*_ values, where *p* < 0,05 (Bonferroni adjusted alpha = 0,05) was accepted as statistically significant. To find out which factors and interactions (partial eta squared values of material type, solution type, and agent type) were affecting the results of Δ*E*_00_, *W*, Δ*W*, TP, and *R*_*a*_ values and to find out the effect size (*R*^2^) of ANOVA tests, tests of between-subject effects were performed. If *p* < 0,05, it means that considered parameter has an effect on the results, and if *p* < 0,001, it means the effect of that parameter is high. *R*^2^ ≥ 80 means the total effect of the parameters included in the measurement is about 80% and the reliability of the statistical analysis and the results is too high.

## 3. Results and Discussion

### 3.1. Results

The mean Δ*E*_00_, *W*, Δ*W*, TP, and *R*_*a*_ values and standard deviations of each group were presented in Tables 2–6.

After staining, only the DW groups of CME and VE showed not perceptible color changes (Δ*E*_00_ < 0,8). The groups which presented clinically unacceptable color changes were TC and RW groups of LU and VE (Δ*E*_00_ > 1,8). Additionally, while there was no statistical difference between Δ*E*_00_ values of TC groups of LU and VE (*p* > 0,05); these groups and RW groups of LU and VE showed statistically significant difference compared to other groups (*p* < 0,05). Regardless of the solution type, there was statistically significant difference between Δ*E*_00_ values of restorative materials where LU showed the highest and CME showed the lowest color changes (*p* < 0,05). Regardless of the material type, there was statistically significant difference between Δ*E*_00_ values according to the solution types, where samples immersed in RW showed the highest and samples immersed in DW showed the lowest color changes (*p* < 0,05).

*W*0 values calculated for CME, LU, and VE were 28,07 ± 1,60, 28,95 ± 0,86, and 38,00 ± 0,78, respectively (*p* < 0,05). DW group of LU showed the lowest *W*1 value and RW group of VE showed the highest *W*1 value. Regardless of the solution type, CME showed the closest color values and VE showed the furthest color values to pure white after staining (*p* < 0,05). Regardless of the material type, samples immersed in DW showed the closest color values and samples immersed in RW showed the furthest color values to pure white after staining (*p* < 0,05). PBO applied RW group of LU showed the lowest *W*2 value and PBO applied TC group of VE showed the highest *W*2 value. Regardless of the solution and bleaching agent type, VE showed the furthest color values to pure white after bleaching (*p* < 0,05). There was no statistically significant difference between LU and CME. Regardless of the material and bleaching agent type, samples immersed in TC showed the furthest color values to pure white after bleaching (*p* < 0,05) and there was no statistical difference between samples immersed in RW and DW. Regardless of the material and solution type, there was no statistically significant difference between the groups according to bleaching agents (*p* > 0,05).

For Δ*W*_*a*_ values, closest coordinates to pure white were observed at PBO applied RW group of LU and the furthest coordinates were observed at OB applied TC group of LU. There was no statistical difference between the groups in terms of Δ*W*_*a*_ values (*p* > 0,05). For Δ*W*_*b*_ values, closest coordinates to pure white were observed at PBO applied RW group of LU and the furthest coordinates were observed at PBO applied DW group of LU. Regardless of the solution and bleaching agent type, CME showed the furthest color coordinates to pure white and LU showed the closest color coordinates to pure white (*p* < 0,05). Regardless of the material and bleaching agent type, samples immersed in DW showed the furthest color coordinates to pure white after bleaching where samples immersed in RW showed the closest color coordinates (*p* < 0,05). Regardless of the material and solution type, there was no statistically significant difference between the groups according to bleaching agents (*p* > 0,05).

According to the baseline TP0 measurements, the most translucent material was LU which was followed by CME and VE, respectively (*p* < 0,05). At the end of the 14th day of staining, there was no statistical difference among the TP1 values (*p* > 0,05). After the bleaching procedure, regardless of the solution and bleaching agent type, LU was the most translucent material and VE was the least translucent material (*p* < 0,05). Regardless of the material and bleaching agent type, TP2 values of samples immersed in DW were the highest and TP2 values of samples immersed in RW were the lowest (*p* < 0,05). There was no statistically significant difference between the effects of 2 bleaching agents on TP2 values of the materials (*p* > 0,05).

According to *R*_*a*_ measurements, regardless from the solution and bleaching agent type, there was a statistically significant difference between the restorative materials with a descending order of *R*_*a*_ as VE, LU, and CME (*p* < 0,05). Regardless of material type and bleaching agent, there was no significant difference between the staining solutions. Regardless of material type and staining solutions, there was no statistically significant difference between the bleaching agents (*p* > 0,05).

According to tests of between-subject effects analysis, the effect size (*R*^2^) of ANOVA tests and *p* values of the parameters were included in the present study and *p* values of the interactions were represented at Table 7.

### 3.2. Discussion

In the initial shade match, color stability of the dental restorations in the oral environment which can determine the life time of the restoration is so important for the patients and clinicians [24]. It has been shown in the literature that the intrinsic and extrinsic factors related to the habits and medical history of the patients [13, 25–27], exposure time, and concentration of the staining agents [25–28] might affect the color stability of the teeth and restorative materials. The alterations in color of the restorative materials may vary due to the composition (photo-initiators, activators, resin matrix, and fillers), physicochemical reactions, hydrophilicity/hydrophobicity, and water sorption of the materials [25–28]. The other factors related to the dental materials are incomplete polymerization, curing time and devices, porosities, oxygen inhibition at the surface, surface treatments like polishing and bond interface, and wear resistance [25–29].

In dental practice, spectrophotometers are commonly used to measure color changes of dental materials more objective than human eye (in 93,3% of cases) and conventional techniques with 33% increase in accuracy [30, 31]. In the present study, color differences (Δ*E*_00_), whiteness index values (*W*^*∗*^), changes in closeness to white values (Δ*W*^*∗*^), and translucency parameters (TP) were calculated by recording *L*^*∗*^, *a*^*∗*^, and *b*^*∗*^ values of the samples on both white and black surfaces according to CIELAB color space with a spectrophotometer.

The current studies showed that CIEDE2000 formula provides better adjustments in color differences by correction of nonuniformity of CIELAB formula [32, 33]. It has been reported that [33–35] CIEDE2000 formula ensures greater correlation between evaluated and perceived color differences and better indicates human perceptibility and acceptability than CIELAB formula for color differences. That was the reason to use CIEDE2000 formula to evaluate the color differences between baseline and after staining, instead of CIELAB formula in the present study. Although at previous studies [34, 36], CIEDE (2 : 1 : 1) was used, due to the lack of data for acceptability and especially perceptibility thresholds of CIEDE (2 : 1 : 1), the use of CIEDE (1 : 1 : 1) formula was preferred for parametric factors in the present study. Bleaching procedures are taking more place at the clinics day by day [13, 14]. To get a faster result, patients can prefer office bleaching which generally uses high concentrations of HP [13, 14]. HP maintains oxidation of the pigments present in enamel and dentin due to its low molecular weight [13–15]. In the present study, 2 office bleaching agents, Perfect Bleach Office+, and Opalescence Boost which includes HP with a concentration of 35% and 40%, respectively, were chosen.

In the present study, A2 shaded 3 restorative materials with 1 mm thickness and with different compositions (a nanohybrid composite resin and 2 ceramic/nanoceramic hybrid CAD/CAM blocks) were tested. The first null hypothesis of this study was partially accepted. After staining, only the DW groups of CME and VE presented not perceptible color changes (Δ*E*_00_ < 0,8). The statistical analysis showed that the material type, staining solution type, and interactions between these 2 parameters had an effect on color changes (*p* < 0,001). According to intragroup comparisons, the RW groups of restorative materials showed the highest and DW groups showed the lowest Δ*E*_00_ values. Although there is no certain proof, the effect of RW may be explained by the low pH, pigments like tannins, and the facilitating effect of alcohol by softening the resin matrix mentioned in previous studies [27, 29, 37]. On the other hand, discoloration by coffee was explained as both adsorption and absorption of colorants into the organic phase of the materials which is probably due to the compatibility of the polymer phase with the yellow colorants of coffee [28]. Only the nanohybrid composite resin (CME) which contains prepolymerized fillers and hydrophobic aromatic dimethacrylate showed color stability in all groups (Δ*E*_00_ < 1,8). As mentioned in a previous study [27], this result may be explained by the effects of sizes and types of the fillers, composition of organic matrix, and polymerization reaction related to composite resins. RW and TC groups of both CAD/CAM blocks presented higher color changes than CME (*p* < 0,05). These results are consistent with the results of previous studies [37, 38]; however, Alharbi et al. [27] reported that CAD/CAM blocks have higher stain resistance compared to the direct composite resins. Water acts as a carrier for staining agents and hydrophilic materials have a higher degree of water sorption resulting in discoloration [27, 28]. According to intergroup comparisons, although there was no statistical difference between TC groups of LU and VE, the statistical difference between RW groups of these materials may be described by the differences at the components and amount of resin matrixes which can affect water sorption and colorant absorption. While VE contains mainly UDMA and TEGDMA 14% by weight, LU contains Bis-GMA, UDMA, Bis-EMA, and TEGDMA 20% by weight. The results of the previous studies showed that although both of the Bis-GMA and TEGDMA are hydrophilic monomers, Bis-GMA causes water sorption ranging from 3% to 6%, while TEGDMA causes water sorption ranging from 0% to 1% [17, 39]. Also previous studies [40, 41] reported that UDMA based materials seemed to be more color-resistant than Bis-GMA because of its low water absorption and solubility characteristics. Consistent with these results, in the present study, VE which has lower amount of resin matrix and different percentages of UDMA and TEGDMA showed lower color changes than LU. On the other hand, although resin matrix of CME contains Bis-GMA with an amount of 22% by weight, this nanohybrid resin showed more color stability compared to the ceramic/nanoceramic hybrid CAD/CAM blocks. This may be explained by the differences of bond interfaces between resin matrix and fillers of these restorative materials.

Currently, to evaluate the whitening effect of bleaching agents, the use of whiteness indices has been suggested [15, 42]. In the present study, CIE whiteness index was used to evaluate the distance of the colors from a nominal white point (*W*^*∗*^ values). Additionally, to evaluate the whitening effect of bleaching agents, closeness to white (Δ*W*^*∗*^) values were used. According to the analysis of these values, the second null hypotheses were accepted. Although all of the restorative materials were A2 shaded, according to *W*0 values, color of CME was the closest color to pure white followed by LU and VE (*p* < 0,05). Considering this result, it may be concluded that the restorations made by these materials with the same thickness may show differences in shade. This may be explained by the differences in absorption and reflection of the light due the composition of the materials like concentration, type, and sizes of filler. According to intragroup comparisons of *W*1 values of restorative materials immersed in DW, only VE showed statistically significant difference with the furthest value to nominal white point. According to intragroup comparisons of *W*1 values of restorative materials immersed in TC and RW, all of the groups showed statistically significant difference where closeness to nominal white from the closest to the furthest was CME, LU, and VE, respectively. These results may be attributed to Δ*E*_00_ color difference values calculated after staining with a conclusion that LU and VE showed darkened color changes while CME showed color stability.

Changes in the closeness to white between baseline and after bleaching (Δ*W*_*a*_) values of the groups did not show any statistical significant difference which means that the effects of bleaching on all of the samples were similar. The negative Δ*W*_*a*_ values mean that, after bleaching, sample has a whiter color than baseline. On the other hand, positive Δ*W*_*a*_ values mean that, after bleaching, sample could not reach the whiteness of the baseline. The results of the present study showed that most whitened group was PBO applied RW group of LU and least whitened group was OB applied TC group of LU. All of the PBO and OB applied DW groups of 3 restorative materials demonstrated negative Δ*W*_*a*_ values. Taking into consideration these results, it may be concluded that the high concentrated bleaching agents can remove the particles causing discoloration and also cause a perceptible color difference at restorative materials by affecting their chemical structure which were not affected with any staining solution. Composite resins generally consist of a polymer matrix mixed with a silanized inorganic filler for bond formation between resin matrix and fillers [43, 44]. On the other hand, while LU consists of a polymer matrix 20% by weight and nanosized zirconia, VE contains polymer matrix 14% by weight and fine structure of feldspathic ceramic. Similar to the color change mechanism of the composite resins, color stability of ceramic/nanoceramic hybrid CAD/CAM materials may be affected by oxidation of amines and leaching of monomers at the resin matrix and oxidation of surface pigments. Also the amount and the type of the resin matrix may be considered as a factor on the color change mechanism of ceramic/nanoceramic hybrid CAD/CAM materials. Especially at the RW groups of LU, whitening effect of the agents was observed more than the other groups. It may be explained by the aforementioned effects of RW as low pH and effects of alcohol like softening the resin matrix. Effects of bleaching agents may increase on the softened matrixes of restorative materials.

There was statistical difference between changes in the closeness to white between baseline and after staining (Δ*W*_*b*_) values of the groups. Similar to Δ*W*_*a*_, negative Δ*W*_*b*_ values mean that after bleaching samples have a whiter color compared to the color reached after staining. Positive Δ*W*_*b*_ values mean that, after bleaching, samples could not reach a whiter color compared to the color reached after staining. With regard to these results, the whitest group was PBO applied RW group of LU, and the least white group was PBO applied DW group of LU. Δ*W*_*b*_ values were higher in TC groups than RW groups which may mean that although RW causes more color changes, it is easier to whiten RW discolorations than discolorations caused by TC. This may also be related to the aforementioned effects of RW on restorative materials and it may be suggested that TC stains the restorative materials more than affecting their chemical structure.

Translucency can be defined as the ability of partially scattering, reflecting, and transmitting the light by an object while the light is passing through it [45]. There are many factors like the composition, shade, and thickness of dental materials which affect translucency [45–48]. The third null hypothesis was partially accepted. For the present study, according to the baseline measurements, the most translucent material was LU and the least translucent material was VE (*p* < 0,05). It has been reported that the light transmission of resin materials is influenced by the difference of refraction index between the filler particle and resin matrix [49]. With regard to this, differences of translucency parameter observed in the present study may be explained by the differences of the chemical structures of the restorative materials and different resin matrixes and different shaped, typed, sized, and concentrated filler particles. The differences between the results of LU and VE at baseline were in agreement with other studies [38, 50], where the thicknesses (1 mm) of the specimens were the same. After staining, there was no statistical difference between the TP1 values (*p* > 0,05). Although the baseline translucency of LU was the highest, the translucencies got similar for all of the restorative materials after staining probably due to the higher discoloration during staining. After bleaching, there were statistically significant differences between the TP2 values according to intragroup comparisons of restorative materials immersed in the same staining solutions (*p* < 0,05). For DW and TC groups, the highest translucency was observed at LU similar to baseline (*p* < 0,05). Considering these findings, while LU became whiter it did not lose its translucency characteristics. This may be considered as an important conclusion that bleaching of restorations made by LU will not cause any consequences about the esthetic appearance.

Surface roughness of restorations has been a major concern for researchers and clinicians, since increase in roughness may enhance the biofilm accumulation resulting in discoloration and secondary caries. The critical *R*_*a*_ value causing biofilm accumulation is determined as 0,2 *μ*m [51, 52]. As mentioned in previous studies [17, 18], surface roughness of the restorations may increase after bleaching due to the composition, exposure procedure of bleaching agents, and the content of dental materials. There are a few techniques for surface analysis like contact stylus tracing, noncontact laser stylus method, compressed air measuring, scanning electron microscopy (SEM), profilometry, and AFM [53]. While most preferred methods are SEM and profilometry, in the present study, the use of AFM was chosen. With the use of AFM, 2D and 3D images can be constructed at the same time and average *R*_*a*_ values are calculated by linearly measured *R*_*a*_ values with minimal sample preparation [7, 54]. Also, SEM needs more steps for preparing samples that can affect the natural structure of the sample and AFM presented more detailed images compared to SEM and profilometry [7, 54].

According to the AFM analysis, the forth null hypothesis was also rejected. After bleaching, it emerged that there were irregularities at surface topography of all specimens. Not only *R*_*a*_ values but also surface topography of CAD/CAM blocks and composite resins presented differences compared to control groups. While the surface alterations were more homogenous at composite resins, CAD/CAM blocks had wider areas for overhangs and recessions. The effect of high energy free radicals liberated from peroxides at the resin-filler interface might cause complete or partial filler-matrix deboning and water uptake leading to increased surface roughness of restorative materials [17, 19, 43]. Additionally, these free radicals and water molecules diffused into resin matrix can attack glass particles, silica, and alumina causing separation of fillers [19, 43]. Although lower *R*_*a*_ values for ceramic/nanoceramic hybrid CAD/CAM blocks were expected because of ceramic components, higher *R*_*a*_ values were observed for CAD/CAM blocks compared to composite resin. VE showed the highest surface roughness values which were also higher than the critical *R*_*a*_ value (*R*_*a*_ > 0,2 *μ*m) for both of the bleaching agents except for PBO applied DW group of VE. Surface roughness values of CME are less than the critical *R*_*a*_ value (*R*_*a*_ < 0,2 *μ*m). Taking into consideration intergroup comparisons for CAD/CAM blocks, PBO applied DW group of LU was the only group showing *R*_*a*_ > 0,2 *μ*m, and oppositely PBO applied DW group of VE was the only group showing *R*_*a*_ < 0,2 *μ*m. These results can be described by the differences at resin matrix of these materials as mentioned before for color changes. Bis-GMA containing dental materials is expected to show higher surface roughness values, but in contrast the results of the present study demonstrated lower *R*_*a*_ values for Bis-GMA containing materials especially at CME groups. The ceramic part of LU mainly consists of nanosized zirconia, while the ceramic part of VE consists of fine structure of feldspathic ceramic. In contrast with the present study, in a previous study [55], where two-body wear, hardness, and surface roughness of polished or brushed different ceramic materials were compared, it was reported that there was no statistical difference between LU and VE. In the same study, [55] a zirconium dioxide ceramic material showed the highest hardness and the lowest two-body wear but no statistical differences at *R*_*a*_ values were found between these materials. Another previous study [56] reported the same results as the present study where brushed LU showed statistically lower *R*_*a*_ values than brushed VE. Zirconia based materials have been suggested to show higher hardness and strength values than feldspathic based materials in other previous studies [55–57]. With that in mind, lower *R*_*a*_ values of LU may be described by the smaller filler size than VE or the structure of LU containing highly cross-linked polymer matrix and zirconia fillers which can be more resistant to the effects of free radicals caused by bleaching agents. Additionally, these findings do not support the hypothesis of Rosentritt et al. [58] reporting that higher filler concentration may contribute to the greater hardness resistance against bleaching agents where VE is the material with the highest weight percentage of fillers.

Taking into consideration these findings, it may be concluded that, in addition to the type and concentration of the components of dental materials and bleaching agents, homogeneity of the composition, bonds between the fillers, and resin matrix are also affecting the chemical reactions between the bleaching agents and dental materials and so color, translucency, surface alterations, and surface roughness.

## 4. Conclusion

Within the limitations of this study, it may be concluded that CAD/CAM blocks showed lower color stability than nanohybrid composite resin. Bleaching agents with high concentrations of HP may provide a reverse effect on color of stained restorative materials and also change translucency values especially after discoloration. Additionally, it may be concluded that bleaching agents with high concentrations of HP may cause surface alterations on dental materials. Ceramic/nanoceramic hybrid CAD/CAM blocks were affected more than composite resin, where VE showed *R*_*a*_ values higher than 0,2 *μ*m which is the critical value for biofilm accumulation. According to these results, it may be suggested that bleaching can be considered as an alternative method for the treatment of stained restorations and high concentrations of HP can be used more safely with composite resins compared to ceramic/nanoceramic hybrid CAD/CAM blocks.

## Figures and Tables

**Table 1:** Composition of the materials which were tested.

Material	Type	Composition	Manufacturer
Clearfil Majesty Esthetic (CME)	Nanohybrid composite resin	Bis-GMA, hydrophobic aromatic dimethacrylates, silanated barium glass filler, prepolymerized organic fillers (78% by weight) Particle sizes: 20 nm–1,5 *µ*m	Kuraray Medical Inc., Tokyo, Japan

Lava Ultimate (LU)	Resin nanoceramic CAD/CAM block	Agglomerated nanoparticles of silica and zirconia (80% by weight), highly cross-linked polymer matrix composed of Bis-GMA, UDMA, Bis-EMA and TEGDMA (20% by weight) Particle sizes: 20 nm silica particles, 4–11 nm zirconia particles	3M ESPE, Bad Seefeld, Germany

Vita Enamic (VE)	Hybrid ceramic CAD/CAM block	Fine structure feldspathic ceramic (86% by weight), resin polymer composed of UDMA and TEGDMA (14% by weight)	VITA Zahnfabrik, Bad Säckingen, Germany

**Table 2:** Mean Δ*E*_00_ values ± standard deviations of the restorative materials at the end of 14th day of staining.

Solution	Material	Δ*E*_00_
Distilled water	CME	0,69 ± 0,44^a^
	LU	1,13 ± 0,43^a^
	VE	0,60 ± 0,29^a^

Turkish coffee	CME	0,93 ± 0,50^a^
	LU	5,93 ± 1,74^b^
	VE	4,93 ± 1,20^b^

Red wine	CME	1,63 ± 0,59^a^
	LU	19,62 ± 2,48^c^
	VE	13,72 ± 3,33^d^

Δ*E*_00_: color difference, CME: Clearfil Majesty Esthetic, LU: Lava Ultimate, and VE: Vita Enamic. Different lowercased letters in the column indicate statistically significant difference between all groups.

**Table 3:** Mean *W* values ± standard deviations of the restorative materials at baseline, after staining and after bleaching.

Solution	Material	*W*0	*W*1	*W*2	
				OB	PBO
Distilled water	CME	28,33 ± 1,55^a,A,B^	27,75 ± 1,36^h,E^	27,17 ± 1,18^p,K^	27,93 ± 1,91^p,K,L^
	LU	28,83 ± 0,88^a,C^	26,81 ± 0,75^h,F^	27,01 ± 0,75^p,M^	26,46 ± 0,71^p,M^
	VE	38,32 ± 0,81^b,D^	37,38 ± 0,62^i,I^	37,95 ± 0,46^q,O,P^	37,50 ± 0,39^q,O,P^

Turkish coffee	CME	28,59 ± 1,71^c,A^	28,88 ± 1,16^j,E^	27,34 ± 1,10^r,K^	29,15 ± 1,52^s,L^
	LU	28,89 ± 0,66^c,C^	35,76 ± 2,14^k,G^	30,29 ± 0,64^s,N^	29,60 ± 0,94^s,N^
	VE	37,95 ± 0,73^d,D^	43,20 ± 1,03^l,J^	38,54 ± 0,46^t,O,P^	38,90 ± 1,03^t,O^

Red wine	CME	27,29 ± 1,28^e,B^	27,76 ± 0,77^m,E^	26,64 ± 1,35^u,K^	26,27 ± 0,91^u,K^
	LU	29,13 ± 1,01^f,C^	39,83 ± 1,63^n,H^	27,49 ± 1,16^u,M^	25,95 ± 1,06^u,M^
	VE	37,73 ± 0,70^g,D^	44,35 ± 1,90^o,J^	38,75 ± 1,18^v,O^	36,89 ± 2,06^v,P ^

*W*0: whiteness index values at baseline. *W*1: whiteness index values after staining. *W*2: whiteness index values after bleaching. CME: Clearfil Majesty Esthetic, LU: Lava Ultimate, and VE: Vita Enamic. Different lowercased letters in the column indicate statistically significant difference according to intragroup comparisons of restorative materials immersed in the same staining solution. Different uppercased letters in the column indicate statistically difference according to intragroup comparisons of the same restorative materials immersed in different staining solutions.

**Table 4:** Mean changes in closeness to white (Δ*W*) values ± standard deviations of the restorative materials after staining and after bleaching.

Solution	Material	Δ*W*_*a*_ (*W*2 − *W*0)		Δ*W*_*b*_ (*W*2 − *W*1)	
		OB	PBO	OB	PBO
Distilled water	CME	−1,07 ± 0,67	−0,49 ± 1,23	−0,51 ± 0,79^a;A^	−0,24 ± 0,81^a;A^
	LU	−1,48 ± 1,15	−2,71 ± 1,44	0,10 ± 0,38^a;B^	0,73 ± 0,53^a;B^
	VE	−0,40 ± 0,82	−0,79 ± 0,61	0,51 ± 0,53^a;F^	0,18 ± 0,66^a;F^

Turkish coffee	CME	−1,04 ± 0,98	0,35 ± 1,48	−1,45 ± 0,80^b;A^	0,18 ± 0,92^b;A^
	LU	1,31 ± 0,78	0,81 ± 1,32	−6,72 ± 1,67^c;C^	−4,90 ± 1,28^c;C^
	VE	0,49 ± 0,56	1,06 ± 1,26	−4,54 ± 1,09^c;G^	−4,41 ± 1,28^d;G^

Red wine	CME	−0,64 ± 0,92	−1,01 ± 1,12	−1,49 ± 1,26^e;A^	−1,10 ± 0,75^e;A^
	LU	−1,82 ± 1,16	−3,00 ± 1,04	−11,65 ± 1,93^f;D^	−14,57 ± 2,22^g;E^
	VE	0,69 ± 1,20	−0,51 ± 2,03	−5,26 ± 1,30^h;G^	−7,80 ± 3,28^i;H^

Δ*W*_*a*_ (*W*2 − *W*0): changes in the closeness to white between baseline and after bleaching. Δ*W*_*b*_ (*W*2 − *W*1): changes in the closeness to white between second week of staining and after bleaching. CME: Clearfil Majesty Esthetic, LU: Lava Ultimate, VE: Vita Enamic, OB: Opalescence Boost, PBO: Perfect Bleach Office+. No significant difference between mean Δ*W*_*a*_ values (*p* > 0,05). Different lowercased letters in the column indicate statistically significant difference according to intragroup comparisons of restorative materials immersed in the same staining solution. Different uppercased letters in the column indicate statistical difference according to intragroup comparisons of the same restorative materials immersed in different staining solutions.

**Table 5:** Mean TP values ± standard deviations of the restorative materials at baseline, after staining and after bleaching.

Solution	Material	TP0	TP1	TP2	
				OB	PBO
Distilled water	CME	16,97 ± 1,13	16,36 ± 1,43	18,00 ± 0,80^a,b;A^	17,06 ± 1,69^a;A^
	LU	20,42 ± 0,78	20,37 ± 0,81	20,44 ± 0,89^b;B^	20,40 ± 0,83^b;B^
	VE	15,94 ± 1,45	15,97 ± 1,25	15,87 ± 1,39^a;E,F^	18,18 ± 6,77^a,b;F^

Turkish coffee	CME	17,04 ± 1,43	16,82 ± 1,27	18,05 ± 0,43^c,d,e;A^	16,43 ± 1,70^c,e,f;A^
	LU	20,77 ± 0,64	18,80 ± 0,95	19,59 ± 0,86^d;B,C^	20,13 ± 0,70^d;B^
	VE	16,02 ± 1,10	13,84 ± 1,39	15,26 ± 1,34^e,f;E,F^	15,89 ± 1,03^f;E^

Red wine	CME	17,65 ± 1,79	15,81 ± 1,76	17,90 ± 0,79^g;A^	17,47 ± 0,48^g;A^
	LU	20,43 ± 0,89	13,27 ± 1,90	16,78 ± 0,96^g,h;C,D^	15,75 ± 0,96^g,h,i;D^
	VE	16,43 ± 0,69	10,00 ± 1,36	14,03 ± 0,97^h,i;E^	13,42 ± 1,32^i;E^

TP0: translucency parameter at baseline. TP1: translucency parameter after staining. TP2: translucency parameter after bleaching. CME: Clearfil Majesty Esthetic, LU: Lava Ultimate, VE: Vita Enamic, OB: Opalescence Boost, and PBO: Perfect Bleach Office+. No significant difference between mean TP1 values (*p* > 0,05). Different lowercased letters in the column indicate statistically significant difference according to intragroup comparisons of restorative materials immersed in the same staining solution. Different uppercased letters in the column indicate statistical difference according to intragroup comparisons of the same restorative materials immersed in different staining solutions.

**Table 6:** Mean *R*_*a*_ values ± standard deviations of the restorative materials after bleaching.

Solution	Material	*R*_*a*_ (*µ*m)	
		OB	PBO
Distilled water	CME	0,013 ± 0,007^a,b;A^	0,008 ± 0,002^a;A^
	LU	0,151 ± 0,079^b,c;B^	0,242 ± 0,097^c;B^
	VE	0,293 ± 0,141^c;C^	0,177 ± 0,045^c;C^

Turkish coffee	CME	0,018 ± 0,01^d;A^	0,012 ± 0,003^d;A^
	LU	0,143 ± 0,087^d,e;B^	0,139 ± 0,06^d,e;B^
	VE	0,257 ± 0,03^e;C^	0,211 ± 0,007^e;C^

Red wine	CME	0,009 ± 0,004^f;A^	0,014 ± 0,005^f;A^
	LU	0,129 ± 0,043^f,g;B^	0,133 ± 0,029^f,g;B^
	VE	0,267 ± 0,034^g;C^	0,225 ± 0,05^g;C^

*R*_*a*_: average surface roughness value measured after bleaching. CME: Clearfil Majesty Esthetic, LU: Lava Ultimate, VE: Vita Enamic, OB: Opalescence Boost, and PBO: Perfect Bleach Office+. Different lowercased letters in the column indicate statistically significant difference according to intragroup comparisons of restorative materials immersed in the same staining solution. Different uppercased letters in the column indicate statistically difference according to intragroup comparisons of the same restorative materials immersed in different staining solutions.

**Table 7:** *R* ^2^ and *p* values calculated for Δ*E*_00_, *W*, Δ*W*, TP, and *R*_*a*_ values according to material, solution, and bleaching agent types and interactions by tests of between subjects effects.

	M	S	A	M and S	M and A	S and A	M, S, and A	*R* ^2^
Δ*E*_00_	0,000	0,000	—	0,000	—	—	—	0,945
*W*1	0,000	0,000	—	0,000	—	—	—	0,961
*W*2	0,000	0,000	0,098	0,000	0,000	0,000	0,604	0,957
Δ*W*_*a*_	0,000	0,000	0,132	0,000	0,002	0,004	0,782	0,541
Δ*W*_*b*_	0,000	0,000	0,408	0,000	0,002	0,000	0,060	0,911
TP1	0,000	0,000	—	0,000	—	—	—	0,820
TP2	0,000	0,000	0,644	0,000	0,283	0,355	0,039	0,568
*R*_*a*_	0,000	0,678	0,339	0,392	0,038	0,964	0,466	0,826

M: material, S: solution, A: bleaching agent, M and S: interactions between material and solution, M and A: interactions between material and bleaching agent, S and A: interactions between solution and bleaching agent, and M, S, and A: interactions between material, solution, and bleaching agent. *R*^2^: effect size, percentage of total effect of the parameters included in the study; *p* < 0,05 means that parameter has an effect on the results. *p* < 0,001 means the effect of that parameter on the results is high.
